# Anti-GABAB Receptor Autoimmune Encephalitis: A Report of a Rare Case in Central America

**DOI:** 10.7759/cureus.68111

**Published:** 2024-08-29

**Authors:** Ana B Santos, Anthony Hong, Isaac Hong, José D Villegas

**Affiliations:** 1 Faculty of Medicine, University of Costa Rica, San José, CRI; 2 Department of Neurology, Hospital San Juan de Dios, San José, CRI

**Keywords:** seizures, paraneoplastic syndrome, gabab receptor, autoantibody, autoimmune encephalitis

## Abstract

Autoimmune encephalitis (AE) is a rare disease. There have been very few reports of anti-GABAB receptor encephalitis, and no case of this subtype has ever been reported in Central America. We present a case of a 21-year-old male patient with an unremarkable previous medical history who was hospitalized because of a new onset of seizures and status epilepticus. Central nervous system infections, neoplastic disorders, cerebrovascular disease, septic and metabolic encephalopathy, and drug toxicity were ruled out. Cerebrospinal fluid (CSF) revealed lymphocytic pleocytosis and oligoclonal bands. Initial head computed tomography (CT) scans with and without contrast were normal, and brain magnetic resonance imaging (MRI) showed no abnormalities. An electroencephalogram showed slow waves and spike waves in the frontal and temporal areas. During hospitalization, encephalopathy progressed, along with seizures and altered mental status requiring mechanical ventilation and admission to the intensive care unit. Intravenous valproic acid and phenytoin for seizure control were given. The unexplained seizures, persisting altered mental status despite the reduction of sedatives, CSF pleocytosis, and oligoclonal bands, along with reasonable exclusion of alternative disorders, suggested AE. The diagnosis was confirmed with positive anti-GABAB1-B2 receptor antibody titers in serum and CSF. A whole-body CT scan showed increased pancreatic head size, but endoscopic ultrasonography ruled out malignancy, and a normal IgG4 range excluded IgG4 disease. The patient received treatment with methylprednisolone, plasmapheresis, and immunoglobulin therapy, with excellent response. The patient has been followed up for seven months, taking immunomodulation with mycophenolate. He is seizure-free with valproic acid and levetiracetam treatment and is receiving cognitive rehabilitation after mild cognitive decline was noted in the psychometric analysis.

## Introduction

Antibody-mediated encephalitis constitutes a group of inflammatory central nervous system disorders that are associated with antibodies against neuronal cell-surfaces proteins, ion channels, or receptors [1]. Although autoimmune encephalitis is a rare disease with an incidence of 0.8/100,000 person-years, it is associated with cognitive decline, epilepsy, behavioral disturbances, and impairment in the level of consciousness [2]. Therefore, prompt diagnosis and treatment lead to improvement or full recovery in most cases [1].

The most common type of autoimmune encephalitis is autoantibodies against the N-methyl-d-aspartate (NMDA) receptor [3]. Other autoantibodies include those directed against the voltage-gated potassium channel complex (VGKC), the α-amino-3-hydroxy-5-methyl-4-isoxazolepropionic acid (AMPA) receptor, the γ-aminobutyric acid (GABA) receptor, anti-dipeptidyl-peptidase-like protein-6 (DPPX, viz. DPP6), and the glycine receptor [4].

We report a case of a patient who was diagnosed with autoimmune encephalitis due to GABAB receptor autoantibodies in Costa Rica, and to the best of our knowledge, it is also the first case reported in Central America.

This article was previously presented as a meeting abstract at the 2024 AAN Summer Conference on July 20, 2024.

## Case presentation

A 21-year-old male consulted the emergency department due to a new onset of seizures and status epilepticus. Past medical history was unremarkable. There was no history of behavioral changes, movement disorder, flu-like symptoms, fever, gastrointestinal symptoms, or history of exposure to zoonotic infections.

Physical exam showed symmetric pupils with normal pupillary light reflex, the neck was supple, and the patient did not have nystagmus, pathological patterns of posture or movement, or abnormal reflexes.

Initial laboratory studies showed complete blood cell count and metabolic panel within normal limits, as shown in Table 1, along with negative Venereal Disease Research Laboratory (VDRL) and HIV serologies. A lumbar puncture was performed and cerebrospinal fluid (CSF) analysis results revealed lymphocytic pleocytosis (100 cells/mm3), normal glucose (70 mg/dL), normal protein levels (25 mg/dL), presence of oligoclonal bands, India ink was negative, and VDRL was not reactive. Culture of cerebrospinal fluid was negative for *Streptococcus pyogenes*.

**Table 1:** Initial laboratory studies of the patient.

Test	Result	Reference range
Hemoglobin (g/dL)	16.6	13.5-17.0
Leucocytes	6070	4000-11000
Platelets	239000	150000-450000
Glucose (mg/dL)	96	70-100
Creatinine (mg/dL)	0.96	0.5-1.5
Total bilirubin (mg/dL)	0.77	0.2-1.2
Sodium (mEq/L)	138	135-145
Potassium (mEq/L)	4.5	3.5-5.3

The molecular panel for meningitis was negative for *Escherichia coli* K1, *Haemophilus influenzae*, *Listeria monocytogenes*, *Neisseria meningitidis*, *Streptococcus agalactiae*, *Streptococcus pneumoniae*, *Cytomegalovirus*, *Enterovirus*, herpes simplex virus 1, herpes simplex virus 2, herpes simplex virus 6, human parechovirus, varicella-zoster virus, and *Cryptococcus neoformans*/*gattii*. Molecular panel for encephalitis was negative for influenza A, SARS-CoV-2, rhinovirus, parainfluenza, and metapneumovirus.

Two head CT scans without contrast and one CT scan with contrast were performed, and neither of them showed space-occupying lesions, bleeding, thrombosis, sinusitis, or mastoiditis.

During hospitalization, encephalopathy progressed with seizures and altered mental status requiring mechanical ventilation and admission to the intensive care unit. IV acyclovir 800 mg three times a day (TID) for 10 days was started empirically, along with valproic acid 400 mg TID and phenytoin 100 mg TID administered orally through a nasogastric tube for seizure control.

**Figure 1:**
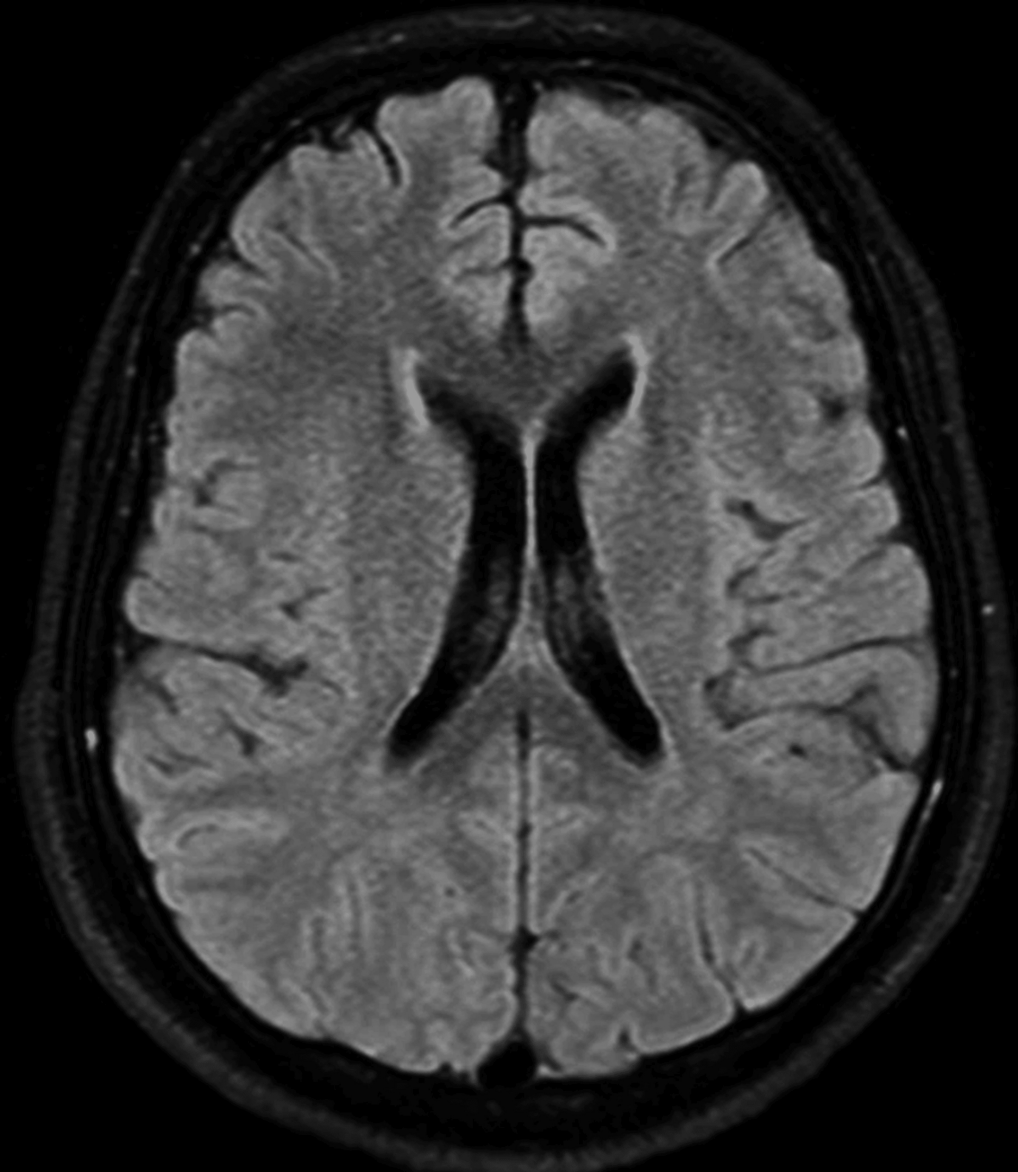
Normal axial brain MRI in fluid-attenuated inversion recovery (FLAIR) sequence. The image shows normal anatomical structures without any signs of abnormal signal changes in the patient.

**Figure 2:**
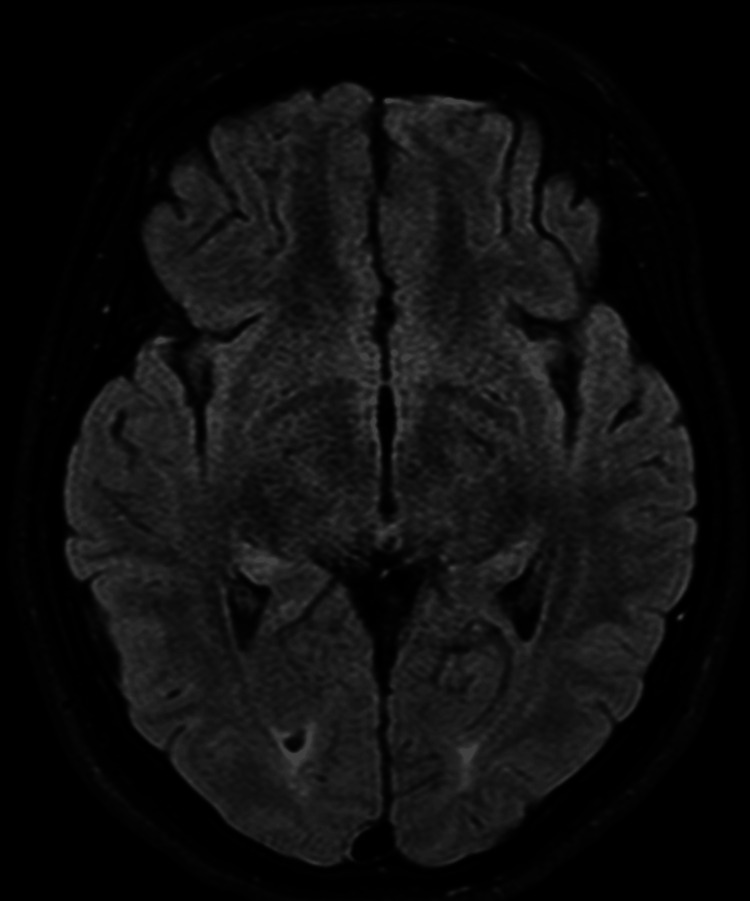
Normal axial brain MRI in fluid-attenuated inversion recovery (FLAIR) sequence. The image shows normal anatomical structures without any signs of abnormal signal changes in this patient.

An electroencephalogram showed slow waves and spike waves in the frontal and temporal area, but a brain magnetic resonance imaging (MRI) showed no abnormalities, as shown in Figures 1, 2.

The patient's persisting altered mental status despite reduction of sedatives and analgesics, in conjunction with the oligoclonal bands found only in CSF and not in serum, which suggests intrathecal IgG synthesis, suggested the diagnosis of autoimmune encephalitis. The diagnosis was confirmed with positive anti-GABAB1-B2 receptor antibody titers in CSF (1:320) and in serum (1:320).

The patient received IV methylprednisolone 1 g/day for five days, five sessions of plasmapheresis, and IV immunoglobulin 25 g/day for five days.

Other studies were performed to rule out malignancy: a testicular ultrasound did not reveal any abnormalities, a CT scan with contrast of the neck and thorax did not show any suspected lesion, and a CT scan with contrast of the abdomen showed an increased size of the head of the pancreas. However, an endoscopic ultrasonography only showed a mildly edematous head of the pancreas, without any other abnormalities. IgG4-related disease was ruled out because serum IgG4 subclass levels were normal.

The patient was discharged with oral prednisolone 50 mg/day for four days, followed by 40 mg/day for seven days, 30 mg/day for seven days, 20 mg/day for seven days, 10 mg/day for seven days, and finally 5 mg/day for seven days. The patient has been followed up for seven months, taking immunomodulation with mycophenolate 500 mg twice per day (BID) orally. He is seizure free with valproic acid 500 mg BID and levetiracetam 1500 mg BID taken orally, and is receiving cognitive rehabilitation after mild cognitive decline was noted in psychometric analysis.

## Discussion

Since anti-GABABR encephalitis was first reported in 2010 by Lancaster et al., the majority of case reports have been from patients residing in developed countries in the northern hemisphere [5]. Vasconcelos et al. reviewed the reported cases of autoimmune encephalitis in Latin America, and there was only one case report of anti-GABA receptor encephalitis with autoantibodies against the GABAA receptor [6,7]. A recent multicenter study from the Brazilian Autoimmune Encephalitis Network reported four cases of anti-GABAB receptor encephalitis [8]. We are now reporting the first case of autoimmune encephalitis with autoantibodies against anti-GABAB receptors in Costa Rica, and to the best of our knowledge, it is also the first one in Central America.

GABA is the main inhibitory neurotransmitter in the central nervous system. The GABAB receptor is a G protein-coupled receptor that consists of two subunits, GABAB1 and GABAB2. The activation of those receptors in the presynaptic and postsynaptic neurons promotes potassium influx and calcium channel inhibition, resulting in the inhibition of neuronal activity. Those receptors are located widely in the brain, predominantly in the hippocampus, thalamus, and cerebellum [9].

Antibodies against GABAB receptors can be triggered by infection, in response to cancer, or by autoimmune mechanisms. Most patients with anti-GABABR antibodies develop autoimmune encephalitis, especially limbic encephalitis [9,10].

Some cohort studies have reported that anti-GABABR encephalitis is more prevalent in males (60-75%), and the median age of onset of the disease is in the 50s (52-61 years of age) [10-12]. Although the case reported is a male, the age of onset of symptoms was 21 years, in contrast with the reported literature.

Anti-GABABR encephalitis typically presents as limbic encephalitis characterized by seizures, cognitive disorders, mental behavioral abnormalities, disturbance of consciousness, and other uncommon clinical syndromes such as cerebellar ataxia and opsoclonus-myoclonus syndrome [11]. The most common initial symptoms are seizures (89.3%-94%), followed by cognitive dysfunction (88%) and psychiatric abnormalities (72%) [12-14]. The initial symptoms in the case reported were seizures, but the patient did not have a history of cognitive disorders or behavioral abnormalities.

There are no diagnostic criteria for encephalitis with anti-GABABR antibodies. The diagnosis is based on classical clinical presentation, imaging, and positive anti-GABABR antibodies in CSF and/or serum [9]. The diagnosis workup in our patient was very complex due to the clinical presentation of seizures without other clinical findings. The initial diagnostic workup ruled out infectious and metabolic diseases, space-occupying lesions in the brain, and bleeding or thrombosis in the central nervous system. There were some key findings reported in the literature that raised suspicion of autoimmune encephalitis: new-onset seizures, oligoclonal bands on CSF, and diffuse slow waves mostly in the frontotemporal lobe [10,12].

Anti-GABABR encephalitis is associated with malignancy, and approximately 50% of patients are diagnosed with small cell lung cancer (SCLC) [12]. Other reported malignancies are thymoma, melanoma, breast carcinoma, rectal carcinoma, multiple myeloma, esophageal carcinoma, sarcomatoid carcinoma (SC), and gastric adenocarcinoma [11]. For that reason, the patient underwent other studies to rule out malignancies, including a CT scan with contrast of the neck, chest, abdomen, and pelvis, endoscopic ultrasonography, and testicular ultrasound, and none of them showed any abnormalities.

Anti-GABABR encephalitis requires a high suspicion and a prompt diagnosis and treatment because it is associated with a high mortality rate within five years. The main predictors of death are old age, the presence of a tumor, the number of complications, and deep venous thrombosis [11]. However, they were not present in our patient.

Patients with non-malignancy-related anti-GABABR encephalitis, as the case presented, have a better prognosis, and they have less incidence of impairment in all the neuropsychological function domains compared with those with comorbid tumors [14,15].

## Conclusions

We present the first case of a patient with anti-GABAB1-B2 receptor autoimmune encephalitis in Central America, with the particular presentation of seizures and status epilepticus without any other clinical symptoms and without any detectable underlying malignancy or infectious disease. It is important to promptly recognize this diagnosis to start early treatment with immunosuppressive therapy with the aim of achieving a better prognosis.
